# Barriers for Access to New Medicines: Searching for the Balance Between Rising Costs and Limited Budgets

**DOI:** 10.3389/fpubh.2018.00328

**Published:** 2018-12-05

**Authors:** Brian Godman, Anna Bucsics, Patricia Vella Bonanno, Wija Oortwijn, Celia C. Rothe, Alessandra Ferrario, Simone Bosselli, Andrew Hill, Antony P. Martin, Steven Simoens, Amanj Kurdi, Mohamed Gad, Jolanta Gulbinovič, Angela Timoney, Tomasz Bochenek, Ahmed Salem, Iris Hoxha, Robert Sauermann, Amos Massele, Augusto Alfonso Guerra, Guenka Petrova, Zornitsa Mitkova, Gnosia Achniotou, Ott Laius, Catherine Sermet, Gisbert Selke, Vasileios Kourafalos, John Yfantopoulos, Einar Magnusson, Roberta Joppi, Margaret Oluka, Hye-Young Kwon, Arianit Jakupi, Francis Kalemeera, Joseph O. Fadare, Oyvind Melien, Maciej Pomorski, Magdalene Wladysiuk, Vanda Marković-Peković, Ileana Mardare, Dmitry Meshkov, Tanja Novakovic, Jurij Fürst, Dominik Tomek, Corrine Zara, Eduardo Diogene, Johanna C. Meyer, Rickard Malmström, Björn Wettermark, Zinhle Matsebula, Stephen Campbell, Alan Haycox

**Affiliations:** ^1^Strathclyde Institute of Pharmacy and Biomedical Sciences, University of Strathclyde, Glasgow, United Kingdom; ^2^Health Economics Centre, University of Liverpool Management School, Liverpool, United Kingdom; ^3^Division of Clinical Pharmacology, Karolinska Institute, Karolinska University Hospital Huddinge, Stockholm, Sweden; ^4^School of Pharmacy, Sefako Makgatho Health Sciences University, Pretoria, South Africa; ^5^Mechanism of Coordinated Access to Orphan Medicinal Products (MoCA), Brussels, Belgium; ^6^Ecorys, Rotterdam, Netherlands; ^7^Department for Health Evidence, Radboud University Medical Center, Nijmegen, Netherlands; ^8^Department of Drug Management, Faculty of Health Sciences, Jagiellonian University Medical College, Krakow, Poland; ^9^Division of Health Policy and Insurance Research, Department of Population Medicine, Harvard Medical School and Harvard Pilgrim Health Care Institute, Boston, MA, United States; ^10^EURORDIS-Rare Diseases Europe, Paris, France; ^11^Institute of Translational Medicine, University of Liverpool, Liverpool, United Kingdom; ^12^HCD Economics, The Innovation Centre, Daresbury, United Kingdom; ^13^KU Leuven Department of Pharmaceutical and Pharmacological Sciences, Leuven, Belgium; ^14^Department of Pharmacology, College of Pharmacy, Hawler Medical University, Erbil, Iraq; ^15^Global Health and Development Group, Imperial College, London, United Kingdom; ^16^Department of Pathology, Forensic Medicine and Pharmacology, Faculty of Medicine, Institute of Biomedical Sciences, Vilnius University, Vilnius, Lithuania; ^17^NHS Lothian, Edinburgh, United Kingdom; ^18^IQVIA, Brussels, Belgium; ^19^Department of Pharmacy, Faculty of Medicine, University of Medicine, Tirana, Albania; ^20^Hauptverband der Österreichischen Sozialversicherungsträger, Vienna, Austria; ^21^Department of Biomedical Sciences, Faculty of Medicine, University of Botswana, Gaborone, Botswana; ^22^Department of Social Pharmacy, College of Pharmacy, Federal University of Minas Gerais, Av. Presidente Antônio Carlos, Belo Horizonte, Brazil; ^23^SUS Collaborating Centre – Technology Assessment & Excellence in Health (CCATES/UFMG), College of Pharmacy, Federal University of Minas Gerais. Av. Presidente Antônio Carlos, Belo Horizonte, Brazil; ^24^Department of Social Pharmacy and Pharmacoeconomics, Faculty of Pharmacy, Medical University of Sofia, Sofia, Bulgaria; ^25^Health Insurance Organisation (HIO), Nicosia, Cyprus; ^26^State Agency of Medicines, Tartu, Estonia; ^27^IRDES, Paris, France; ^28^Wissenschaftliches Institut der AOK (WIdO), Berlin, Germany; ^29^EOPYY-National Organization for the Provision of Healthcare Services, Athens, Greece; ^30^School of Economics and Political Science, University of Athens, Athens, Greece; ^31^Department of Health Services, Ministry of Health, Reykjavík, Iceland; ^32^Pharmaceutical Drug Department, Azienda Sanitaria Locale of Verona, Verona, Italy; ^33^Department of Pharmacology and Pharmacognosy, School of Pharmacy, University of Nairobi, Nairobi, Kenya; ^34^Division of Biology and Public Health, Mokwon University, Daejeon, South Korea; ^35^UBT - Higher Education Institute, Prishtina, Kosovo; ^36^Department of Pharmacology and Therapeutics, Faculty of Health Sciences, University of Namibia, Windhoek, Namibia; ^37^Department of Pharmacology and Therapeutics, Ekiti State University, Ado-Ekiti, Nigeria; ^38^Norwegian Institute of Public Health, Oslo, Norway; ^39^Agency for Health Technology Assessment and Tariff System (AOTMiT), Warsaw, Poland; ^40^HTA Consulting, Cracow, Poland; ^41^Ministry of Health and Social Welfare, Banja Luka, Bosnia and Herzegovina; ^42^Department of Social Pharmacy, Faculty of Medicine, University of Banja Luka, Banja Luka, Bosnia and Herzegovina; ^43^Public Health and Management Department, Faculty of Medicine, “Carol Davila”, University of Medicine and Pharmacy Bucharest, Bucharest, Romania; ^44^National Research Institution for Public Health, Moscow, Russia; ^45^ZEM Solutions, Belgrade, Serbia; ^46^Health Insurance Institute, Ljubljana, Slovenia; ^47^Faculty of Medicine, Slovak Medical University in Bratislava, Bratislava, Slovakia; ^48^Drug Territorial Action Unit, Catalan Health Service, Barcelona, Spain; ^49^Vall d'Hebron University Hospital, Fundació Institut Català de Farmacologia, Barcelona, Spain; ^50^Department of Medicine Solna, Karolinska Institutet and Clinical Pharmacology Karolinska University Hospital, Stockholm, Sweden; ^51^Department of Healthcare Development, Stockholm County Council, Stockholm, Sweden; ^52^Raleigh Fitkin Memorial Hospital, Manzini, Swaziland; ^53^Division of Population Health, Health Services Research and Primary Care, Centre for Primary Care, University of Manchester, Manchester, United Kingdom; ^54^NIHR Greater Manchester Patient Safety Translational Research Centre, School of Health Sciences, University of Manchester, Manchester, United Kingdom

**Keywords:** managed entry, health policy, pharmaceuticals, financing, cancer, orphan diseases, new models

## Abstract

**Introduction:** There is continued unmet medical need for new medicines across countries especially for cancer, immunological diseases, and orphan diseases. However, there are growing challenges with funding new medicines at ever increasing prices along with funding increased medicine volumes with the growth in both infectious diseases and non-communicable diseases across countries. This has resulted in the development of new models to better manage the entry of new medicines, new financial models being postulated to finance new medicines as well as strategies to improve prescribing efficiency. However, more needs to be done. Consequently, the primary aim of this paper is to consider potential ways to optimize the use of new medicines balancing rising costs with increasing budgetary pressures to stimulate debate especially from a payer perspective.

**Methods:** A narrative review of pharmaceutical policies and implications, as well as possible developments, based on key publications and initiatives known to the co-authors principally from a health authority perspective.

**Results:** A number of initiatives and approaches have been identified including new models to better manage the entry of new medicines based on three pillars (pre-, peri-, and post-launch activities). Within this, we see the growing role of horizon scanning activities starting up to 36 months before launch, managed entry agreements and post launch follow-up. It is also likely there will be greater scrutiny over the effectiveness and value of new cancer medicines given ever increasing prices. This could include establishing minimum effectiveness targets for premium pricing along with re-evaluating prices as more medicines for cancer lose their patent. There will also be a greater involvement of patients especially with orphan diseases. New initiatives could include a greater role of multicriteria decision analysis, as well as looking at the potential for de-linking research and development from commercial activities to enhance affordability.

**Conclusion:** There are a number of ongoing activities across countries to try and fund new valued medicines whilst attaining or maintaining universal healthcare. Such activities will grow with increasing resource pressures and continued unmet need.

## Introduction

Spending on medicines is a concern across all countries due to changing demographics and lifestyles leading to increased medicine use, stricter targets for treating patients, rising patient expectations and the continued launch of new premium priced medicines (1–6). The costs of medicines are a particular issue in low and middle income countries (LMICs) where they can account for up to 70% of total healthcare expenditure (7, 8), exacerbated by high and growing prevalence of non-communicable diseases (NCDs) such as diabetes and hypertension. The growing prevalence of NCDs in LMICs leads to issues of affordability (9), with funding of medicines for patients with cancer and immune diseases such as rheumatoid arthritis a particular challenge due to their costs (10–13). High income countries are also struggling to fund new premium priced medicines in all or some populations (3, 14). This situation will worsen with continuing unmet need (4) leading to continued debates regarding the possible funding of new high priced medicines in different disease areas.

Total expenditure on medicines among OECD countries in 2015 was over US$800 billion and rising as a result of appreciable increases in expenditure on new medicines for hepatitis C and in oncology (15). Concerns with the potential budget impact of second generation direct acting antivirals (DAAs) resulted in restrictions on their use despite their undoubted effectiveness (16–19). There were also intense price negotiations across countries with for instance the authorities in France initially faced with the cost of sofosbuvir at 756 times its cost of production, i.e., potentially over 99.8% gross profit, before negotiations (20). However, prices in some countries, e.g., Egypt, were discounted up to 99% from the US prices to support affordability (16, 17, 20). Overall, prices were substantially lower for second generation DAAs in LMICs to help with affordability (21).

The increased expenditure on cancer medicines has been augmented by prices for new cancer medicines rising by up to 10-fold during the last decade (22, 23). As a result, expenditure on medicines for patients with cancer now dominate pharmaceutical expenditure in developed markets (24). The increasing prevalence of patients with cancer, coupled with rising prices (25–28), has seen world-wide sales of medicines for cancer reach $107billion in 2015, an increase of 11.4% since 2014 (29). Expenditure on cancer medicines will rise further with new cancer cases anticipated to rise to 21.4 million per year by 2030 (22, 30) coupled with the appreciable pipeline of new potential premium priced oncology drugs with more than 500 companies actively pursuing new oncology medicines in over 600 indications (29).

The increasing expenditure on new premium priced medicines in recent years, including those for cancer, is despite their questionable therapeutic value, with most new medicines revealing limited or no health gain vs. existing therapies when appraised by independent drug information journals (Table 1) (33–37).

**Table 1:** Percentage ratings for new medicines and new indications introduced in France between 2010 to 2015 [Adapted from (2, 31, 32)].

**Prescrire ratings/criteria**	**2010**	**2011**	**2012**	**2013**	**2014**	**2015**
Total number of new medicines/new indications	97	92	82	90	87	87
Innovative medicine/ real therapeutic advance	1%	0%	1%	0%	3%	3%
Offers an advantage over current standards	3%	3%	4%	7%	6%	6%
Possibly helpful, minimal or no clinical advantage compared to existing standard treatments	73%	72%	68%	66%	58%	67%
Others including not being seen as acceptable due to known or suspected serious adverse events as well as uncertain, unproven or limited effectiveness. In addition, judgement reserved regarding the possible level of innovation due to insufficient data available	23%	25%	27%	27%	33%	24%

This is certainly the case for new cancer medicines as there is limited health gain for most alongside uncertain evidence (25, 38–42). However, high prices have been facilitated by pharmaceutical companies seeking orphan status for their new cancer medicines. This is despite the cost of goods for a number of new cancer medicines as low as 1% of originator prices (43, 44).

There have also been concerns with funding for new medicines for patients with orphan diseases given increasing prices and uncertain evidence (33, 34, 45, 46), again exacerbated by the emotive nature of the disease area (37). In view of the number of medicines for orphan diseases currently available and in development, it is likely that global spending on orphan medicines will reach US$178billion per year by 2020 (47), equalling the amount spent on medicines for patients with cancer. There may though be some overlap with a number of new antineoplastic medicines designated as orphan status. However, concerns with the requested prices for medicines for orphan diseases, and their potential budget impact, has to be balanced against incentives for pharmaceutical companies to develop new medicines to address unmet need (48, 49). We are also aware that the prices for a number of medicines for orphan diseases are sustainable (50), and that sales of medicines for orphan diseases can be limited, e.g., sales of Pfizer's Elelyso® (taliglucerase alfa) for type 1 Gaucher disease had a net revenue of only US$48million in 2016 (47). We have also seen the establishment of European Reference Networks for patients with rare diseases to accelerate research in the area of orphan diseases, which coupled with a greater role of patients and registries, could help with appropriate pricing models (47, 51, 52). However, this remains to be seen.

Adaptive pathways have also been proposed to accelerate the introduction of innovative medicines in Europe (53, 54). However, there are considerable concerns among European payers in this regard, and these will continue.

The increasing impact of personalized medicine within a number of therapeutic fields is also a major challenge with regard to the developmental chain for new medicines. This includes evidence generation through clinical research, optimization of novel treatment approaches, adaptation of tools for assessment, reimbursement mechanisms, costs, and monitoring of outcomes (55–58). For example, the use of health technology assessments (HTA) to reveal the added values of treatments in personalized medicine is challenging, which in turn may well have an impact on reimbursement processes. There are also concerns with the funding of new gene therapies at ~US$1.3million per dose, although limited sales to date (47).

Concurrent with these developments, we have seen health authorities instigate educational and other activities as part of developing a comprehensive model to better manage the entry of new medicines where there have been concerns with their potential safety and/ or expenditure in routine clinical care. This was the case with dabigatran with increased acquisition costs over warfarin coupled with concerns with potentially excessive bleeding and deaths arising from inappropriate use, exacerbated by issues with the commercial activities of the company (3, 59, 60). There was typically no excessive bleeding and no excessive deaths in countries and regions that had instigated educational and other activities to enhance the appropriate use of dabigatran post launch (59, 61).

We are also seeing the growth in managed entry agreements (MEAs) and other mechanisms to lower the price of new medicines to enhance their chances of reimbursement (62–66). In addition, the development of multiple criteria decision analysis (MCDA) tools for valuing new medicines especially those for orphan diseases (2, 67–72).

Alongside this, health authorities across countries have instigated a variety of measures and initiatives to improve prescribing efficiency to release valuable resources. These include measures to direct physicians toward prescribing well proven and effective medicines, enhance the prescribing of generics versus originators and patented products in a class and biosimilars, as well as actively pursue disinvestment of technologies where pertinent (73–79). Strengthening pro-generic and biosimilar policies, as well as increasing transparency, are especially important in LMICs to enhance access to medicines (80, 81). The prescribing of generics is growing across countries as more originators lose their patents, helped by prices of generics as low as 2% of originator prices (82–85). However, we are aware that any quality, safety and efficacy concerns with generics need to be addressed before savings can be fully realized (86, 87).

Given ongoing concerns and barriers to the funding of new medicines with increasing resource pressures, the aim of this paper is to consider potential ways to optimize the use of new medicines balancing rising costs with increasing budgetary pressures. This includes potential approaches to the funding of research and development (R & D) for new medicines. The situation in LMICs can be more challenging with issues of affordability and access even to essential medicines. As a result, we hope to stimulate future debates in this crucial area.

## Methods

We present a narrative review of pharmaceutical policies and implications, as well as possible developments, based on key publications and initiatives known to the co-authors who are experts in this research and practice area. This is principally from a health authority perspective as many of the co-authors are senior level decision makers and advisers in their country. We have used this approach before in a number of previous publications to provide direction and stimulate debate (2, 33, 55, 75, 88–91).

We do not discuss specifically pricing and reimbursement policies across countries as this has been undertaken by others (34, 92–94). We also do not discuss ongoing initiatives to increase the prescribing of low cost generics or biosimilars, or disinvestment practices, as these initiatives have also already been covered by the co-authors and others in a number of published studies (75, 76, 78, 79, 95–98). We also do not discuss issues of adherence to medicines, which is of particular concern with medicines for patients with NCDs, with non-adherence negatively impacting on morbidity, mortality and costs (99–104), as these issues are outside the scope of this paper. We are also aware of access programmes especially in African countries for key medicines for infectious diseases and NCDs (9, 105, 106). However, again this is outside the scope of this paper although this will be briefly mentioned when discussing different pricing approaches as well as potential ways forward.

## Results—Ongoing Initiatives

There are a number of ongoing approaches to enhance the funding for new valued medicines especially among higher income countries given ongoing budgetary constraints. These include developing new models to optimize their managed entry including issues of MEAs and post launch registries; developing and testing MCDA approaches; and developing new financing approaches. These will now be discussed in detail building on recent publications to give future direction with a particular emphasis on medicines for cancer and orphan diseases (34, 94, 107–111).

### New Models to Optimize the Managed Entry of New Medicines

**Figure 1:**
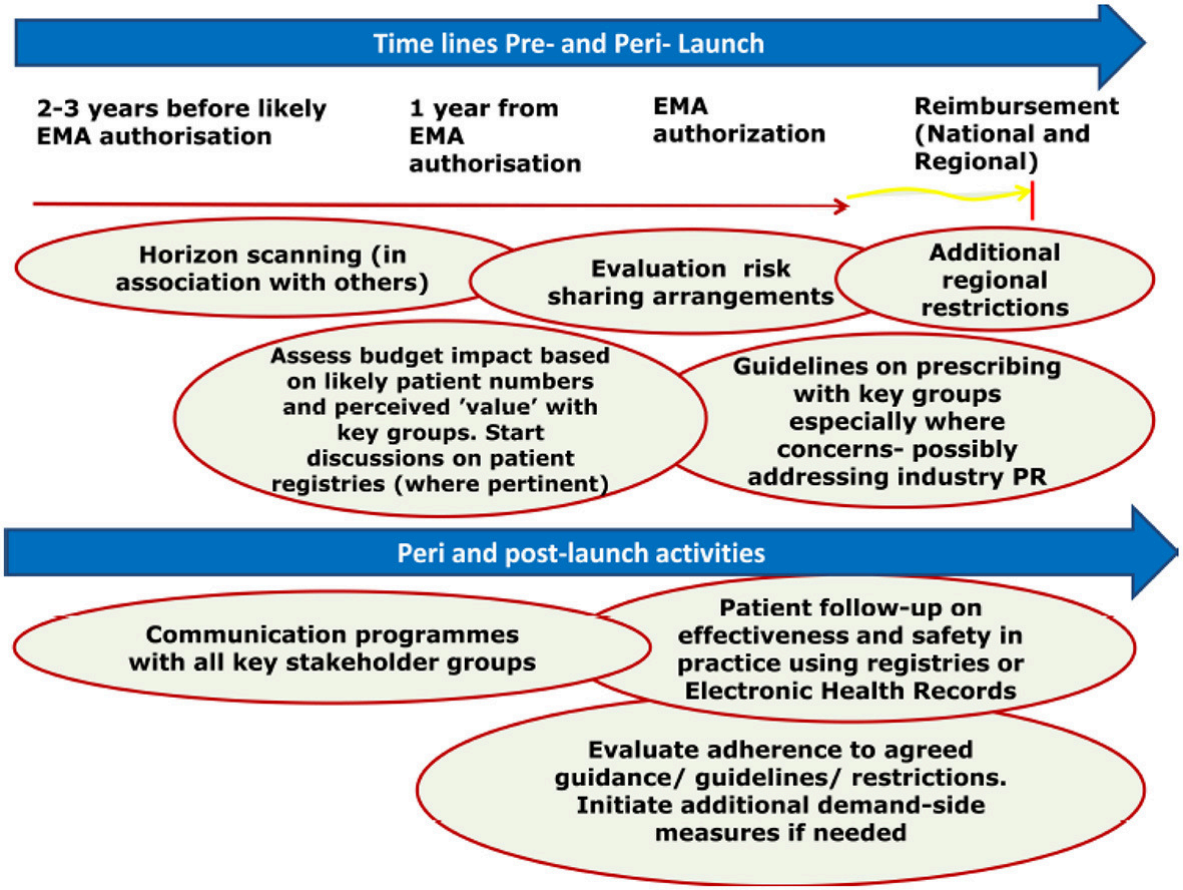
Ongoing model to optimize the managed entry of new drugs across Europe incorporating national and regional stakeholder groups (Reproduced with kind permission of Frontiers in Pharmacology) (3, 59).

Payers and their advisers across Europe and wider have developed new models to optimize the managed entry of new medicines in response to ever increasing costs of new medicines, which build on initial initiatives in Stockholm, Sweden, and across Europe (112, 113). The models have subsequently been refined based on activities surrounding the launch of dabigatran to help prevent potential excessive bleeding (Figure 1) (3, 59), with further iterations since then. These models are typically based on three pillars: pre-, peri-, and post launch activities (3, 33, 34, 114–116).

We are aware that there is appreciable variation in the uptake and utilization of new medicines across countries as seen for instance with physicians in the UK typically more conservative in adopting new medicines than colleagues in Italy, Spain or the US (117–120). Generally, critical factors in the uptake and utilization of new medicines across sectors include issues of involvement in clinical trials, influence and impact of drug and therapeutic committees (DTCs), cost of the new medicines to hospitals including potential budgets and any discounts, as well as pharmaceutical industry and patient pressures (115, 117, 121–128). Introducing a more structured approach (Figure 1) seeks to address a number of these issues and concerns.

Pre-launch activities include horizon scanning and budgetary planning, with peri-launch activities including assessing the role and value of new medicines using robust methodologies as well as appraising proposed MEAs (34, 115, 129). Post launch activities including evaluating the effectiveness and safety of new medicines in routine clinical practice via registries and other approaches as well as assessing prescribing against agreed guidelines (Figure 1). The following sub-sections contain further details.

#### Pre-launch Activities

Pre-launch activities include horizon scanning for new medicines to prepare for the future. Horizon scanning for medicines is defined as “*identifying new medicines or new uses of existing medicines that are expected to receive marketing authorization from the Regulatory Authority in the near future and estimating their potential impact on patient care*” (113, 130, 131), with activities growing across countries and regions (131–137). This includes recent proposals for cross country collaboration in Horizon Scanning (137). A recent publication from Sweden has shown good sensitivity for such analyses especially in the oncology/immunomodulating areas (138).

**Figure 2:**
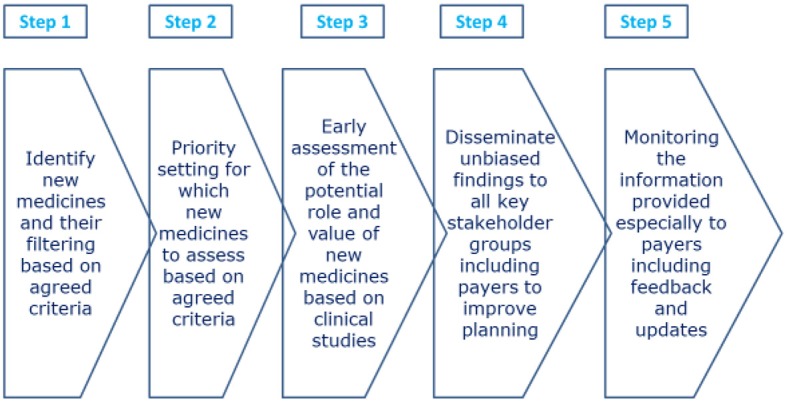
Sequenced approaches for Horizon Scanning activities [adapted from (116, 133, 140, 141)].

Horizon scanning activities can include budget forecasts in all or some populations to help fund their utilization within agreed budgets (115, 116, 136, 139). They typically consist of a number of sequenced approaches (Figure 2).

Typical prioritization criteria for selecting which medicines to review as part of horizon scanning activities include (116, 133, 136, 140):

- Number of potential patients for the new medicine and disease severity
- Intended use of the new medicine, i.e., add-on to existing medicines, a replacement for existing treatments or an innovative approach
- Likely health gain of the new medicine vs. existing standards including any potential safety issues, i.e., potential to critically improve patient outcomes
- Current clinical development status, i.e., Phase II or III
- Estimated budget impact based on likely costs—especially if likely comparators will soon lose their patent
- Potential for off-label use and its budget impact
- Potential to result in re-organization of healthcare for the disease area
- Possible high media/ public interest.

Horizon scanning activities often start up to 24 to 36 months before likely European Medicines Agency (EMA) marketing authorization. They include providing data to regional and national payers on the findings from Phase II studies and ongoing Phase III trials, with more complete data provided nearer marketing authorization by regulatory agencies such as the EMA (133, 136, 137). However, providing horizon scanning data just before marketing authorization may be too late for some healthcare systems to effectively react with increased budgets for new valued medicines (142).

We are also seeing the growth in budget impact analyses (BIAs) to help estimate the potential financial implications of new technologies and their possible impact on future spending (116, 143). The key components of any BIA should include the following: (i) the budget holders' perspective; (ii) the defined time horizon for the analysis, e.g., up to 36 months post launch; (iii) clear identification of the setting; (iv) budget estimates expressed as undiscounted cost differences between any new medicine and the current situation; (v) estimates taking into account potential trade-offs in healthcare resources based on possible variations in the effectiveness of the new medicine; and (vi) sensitivity analyses taking into account the uncertainty surrounding future care (116, 144). Drug utilization studies using patient level data enhance the robustness of forecasted budgets through reducing uncertainty as seen for instance in Brazil when documenting the cost of atypical antipsychotics in the public healthcare system (145). Robust patient level data is also used to develop forecasts for the potential spend on new medicines in Stockholm County Council, Sweden, helped by expert groups and interaction with pharmaceutical companies (136, 139).

It is increasingly recognized that a more integrative approach is needed in the future to enhance the usefulness of peri-launch activities, including increased use of HTA techniques, which will require new skills (142). In addition, considering smart data systems and greater international collaboration to improve the efficiency and usefulness of horizon scanning activities and outputs (142).

Independent willingness-to-pay and other preference elucidation studies can also be conducted at this time to help authorities in their pricing negotiations, especially if such data is taken into account during pricing negotiations as seen currently in Brazil (146, 147).

The development of any potential quality indicators to improve the prescribing of the new medicine post launch should also ideally be considered pre-launch and not several years after launch, which has typically been the case in the past (148). The same applies to the development of any patient registry for new medicines especially for patients with orphan diseases (3, 47), which can be part of ongoing measures to enhance physician adherence to any prescribing guidelines. Specific biomarkers to better target patients for new therapies should also be highlighted given the growth in pharmacogenomics (55).

#### Peri Launch

Countries have typically adopted different approaches to the pricing and reimbursement of new medicines (34, 92, 94, 141, 149, 150). These can be classified into those that first assess the level of innovation of new medicines against existing standards before negotiating prices, such as Austria, France, and Germany (34, 92, 115, 151, 152), as opposed to those countries that base reimbursement and funding decisions on economic criteria such as cost/quality adjusted life year (QALY), with or without threshold levels, as seen for instance in Belgium, Sweden and the United Kingdom (33, 34, 92, 110, 153, 154). Other countries apart from European countries that also increasingly include economic criteria in their pricing and reimbursement decision making include Australia, Brazil, Canada, Korea, and New Zealand (2, 92, 146, 155, 156). Currently though, only a minority of countries such as Poland, Slovakia, and the United Kingdom use economic principles to set threshold levels (63, 92), although some countries such as Belgium provide guidance (110). There are suggestions by some authors that any proposed levels should be lowered for long term sustainability (157). However, the lack of standard methodologies for determining possible thresholds, with the WHO recently stating it does not recommend three times GDP per capita as being relatively cost effective (158), appears to have provided manufacturers with a means to justify higher prices (159).

Whichever method is used, all incorporate approaches to assess the value of new medicines based on HTA principles and techniques with variable levels of uncertainty, with the concept of value based pricing (VBP) increasingly utilized (34, 66, 141, 160). However, whilst VBP is a well-established concept, there is no universal definition and its implementation has been challenging (129, 158, 161, 162). Having said this, VBP typically means the use of strategies designed to link prices of a perceived medicine with its perceived value (153, 158, 163), with issues such as the cost-effectiveness of a new medicine just one of the key considerations for reimbursement alongside issues of affordability and budget impact (66, 158, 161). Such considerations are particularly important in LMICs (164).

As a result of increasing resource pressures, we are likely to see the continued role of robust comparative effectiveness analysis along with BIAs in reimbursement decisions (158), especially with limited perceived innovation with most new medicines (Table 1). This will also require more independent BIAs being performed to help assess issues of affordability of new medicines given concerns with bias if BIAs are performed by commercial organizations (165). In addition, acknowledging differences between payers and companies, with companies typically seeking prices that may not be consistent with VBP on the basis that their new medicine, such as those for cancer and orphan diseases, should be treated differently (36, 49, 153). These concerns can be eased by companies providing data to suggest in which sub-populations their new medicine will provide most benefit, which should be aided by the increasing availability of biomarkers (55, 166), as well as more closely aligning requested prices with the additional health gain of their new medicine (25, 39, 49). The alternative is for healthcare sectors to impose restrictions themselves on the use of new and expensive medicines to stay within agreed budgets (115), which may be more arbitrary potentially adversely impacting on care provision. Alongside this, prices and/ or discounts for patented medicines will have to be increasingly re-evaluated as comparators become available as either low cost generics or biosimilars.

We are also likely to see increased cross border and regional collaboration among countries to enhance their pricing negotiations (46, 167–170). The number of MEAs, also called risk sharing arrangements, will also grow with pharmaceutical companies wishing reimbursement for their new medicines (65). This is because reimbursement is essential in most countries otherwise there will be limited funding and utilization of any new medicine.

There is also an ongoing proposal from the European Commission to strengthen joint cooperation on HTA beyond 2020 (171–173). Whilst such an approach is welcome to reduce duplication of activities, which is especially important among European countries with limited resources, a number of concerns have been identified with the proposal as originally set out. These include making sure that any European Coordination Group has the final say on any joint assessment report, which includes safety data and relative effectiveness assessments, rather than the Commission (as recommended in the proposal). This is important since there are concerns with pushing for acceptance of early assessments with greater uncertainty with available data extending from concerns with adaptive pathways (53, 54). In addition, there must be continued flexibility to allow national decision makers to be actively involved when using any joint report for their reimbursement decisions given ongoing differences in the availability and affordability of medicines between European countries (171).

Other ways to influence prices of medicines, and hence their affordability, include revising wholesaler and pharmacy mark-ups where needed (33, 174). Wholesaler margins typically vary between 2 and 8% of the pharmacy retail price in Europe, although mark-ups as high as 24% have been seen for a small number of medicines (174). For instance, the mark-up in Croatia was 8.5% of ex-factory prices, and in Greece there were up to 130 wholesalers at one stage vs. for instance twenty in the UK and three in Denmark with mark-ups as high as 7.8% before falling to 5.4% (175, 176). Pharmacy mark-ups have typically ranged between 18 and 25% among European countries, although mark-ups have been as low as 12% and as high as 50% (174). Whether a mark-up is based on a percentage of the base price (which can result in unduly high or low absolute amounts) or a flat fee, or a combination of both, is better, is an ongoing discussion across countries. Given the current political situation within countries, this has to be decided at the national level by each Member State in the European Union and other countries. Such debates are increasing especially with the continued launch of expensive new medicines in ambulatory care coupled with the increasing availability of low cost generics.

##### Managed entry agreements

MEAs can be divided into (a) financial schemes typically involving discounts, rebates, or price volume agreements and (b) performance based schemes including outcome guarantee schemes (63, 91, 110, 150). Schemes involving (usually confidential) discounts, rebates, or price: volume agreements, are increasingly seen in practice as they are easier to administer (150, 155, 177). Outcome or performance based agreements are seen as more problematic since they increasingly require robust and sophisticated systems to collect the data and many confounders may influence the outcomes in real life (63, 66, 110). This can potentially be addressed through clear definitions of the data to be collected, open and agreed standards for outcomes to be measured, clear and transparent decision making rules, openness of the information collected, as well as clarifying early in the process who owns and funds any patient registries that will be used (108). Such schemes can be linked to coverage with evidence schemes; however, these can also be problematic in Europe with limited incentives for companies to collect additional data and difficulties with delisting new medicines from reimbursement lists based on their value rather than safety (108, 110). Overcoming the latter may well require strengthening of health authorities' abilities to uphold negative funding decisions (108). In addition, in the case of oncology, performance based schemes have typically been based on surrogate markers rather than clinically relevant outcome measures (178). This can be a concern if there is no clear link between the surrogate markers used and improved overall survival, which is a key goal of treatment (25, 179, 180). As a result, we are now seeing disillusionment and an ensuing decline in the use of this particular type of MEA (181). However, they have proven beneficial under the right circumstances (182), and we may see their use increasing again as the use of “real world” evidence generation grows (66, 183, 184). Outcome based schemes may also achieve lower prices in practice than indication based pricing schemes with their many challenges (185).

Overall despite these concerns, we are likely to see continuing growth in MEAs to enhance access to new medicines, especially new oncology medicines, as countries struggle to stay within budgets with ever increasing prices (62, 65, 107, 150). However, there is increasing agreement that MEAs should not become the norm for introducing new medicines (111). The need for MEAs is symptomatic of unsustainable prices and their use fosters a lack of transparency. Overall, new medicines should be priced at sustainable levels for all key stakeholder groups, and the use of MEAs should be the exception rather than the norm aimed particularly for new medicines with anticipated high expenditure and uncertain longer-term clinical benefits (111).

Having said this, experience in implementing MEAs does vary substantially across Europe. It is recognized that there is considerable scope for countries to share their experiences recognizing though that publications assessing such schemes may be limited due to data protection concerns (63, 150). It is essential going forward that all key stakeholder groups should also ask themselves whether confidential discounts within public health care systems should be continued (63), and whether legislative measures could be introduced to force companies to disclose discount levels (167). This is important as it is not clear who is really benefiting from existing MEA schemes especially in countries where there are substantial co-payments for medicines based on list rather than discounted prices (63). In the future, countries need to establish clear objectives for any agreed MEA as well as seek to introduce a monitoring framework alongside assessing the financial burden of their implementation. Such information will help health authorities fully evaluate the role of MEAs in the future.

##### Indication-based pricing for new medicines

We are aware that there are proposals to incentivise pharmaceutical companies to invest in ongoing research for new indications for existing treatments to reduce the reliance on new medicines (186). There have also been calls for indication specific pricing to expand the use of existing medicines (187, 188). However, there are concerns about the availability of robust information systems to track this, similar to concerns with outcome based MEAs (188).

In addition, some companies already promote the off-label use of their medicines, as well as increase the number of indications post launch, to extend their patent life, and hence profitability (189–191). This is after they have secured optimal prices for their new medicine based on the greatest value in a given population. Extending the patent life has been enhanced by the issuing of supplementary protection certificates (SPCs) (192).

However, such activities can cause concern especially in countries such as the UK with high international non-proprietary name (INN) prescribing once the first indication loses its patent. Doctors in the UK were threatened with legal action if they prescribed pregabalin by INN name for neuropathic pain rather than the originator, with the neuropathic pain indication still patent protected (190). This is because high INN prescribing is routine in the UK apart from a minority of situations (193, 194).

These issues and concerns will need to be resolved before indication-based pricing becomes a realistic option. However, this has to be balanced against decreasing times for patent protection with stricter regulatory requirements for new medicines which SPCs can help address (192).

##### Differential pricing between countries as well as cross border collaboration with pricing negotiations

An alternative to confidential discounts to enhance the chances of reimbursement for new premium priced medicines, especially among LMICs where affordability is an issue, is variable pricing based on countries' ability to pay (111, 195). The European Pharmaceutical Industry Federation (EFPIA) recently considered the possibility of tiered pricing for European countries struggling to fund new medicines, although this has not been taken further (2). This may be because such considerations will be complex given for instance free movement of services and goods in Europe (196) and potentially difficult within the confines of external reference pricing despite its many challenges and concerns (111, 197–199).

There are also concerns that such strategies may decrease transparency between pharmaceutical companies and health authorities (200), that such arrangements amount to developed countries subsidizing medicines for LMICs, and that companies may not have strong incentives to re-evaluate tier prices in the absence of competition (195). There may though be situations where tiered pricing is an option to address affordability issues for new valued medicines; however, there is a need to stimulate competition in the long term to ensure continued availability and affordability of new medicines (2, 195).

Other approaches include LMICs taking advantage of public health intellectual property flexibilities, and LMICs working with pharmaceutical companies and others to make sure essential medicines are made routinely available (30, 195, 201). Private public partnerships between government and the pharmaceutical industry are growing among LMICs to decrease costs and increasing availability. In South Africa, the Biovac Institute will in the future locally manufacture the hexavalent vaccine, which protects against six life-threatening infections, with an estimated medium term saving of 15% (202, 203). Partnerships are also growing in Brazil to increase access to biosimilars and medicines for HIV (204, 205). In addition, pharmaceutical companies are making medicines for patients with NCDs available for as little as US$1/patient/ month to address issues of affordability (9). This is happening in Kenya, and we will be monitoring the findings (105, 206). Janssen has also recently signed an agreement to lower the prices of medicines for TB in South Africa (106). It is likely such arrangements will grow in the future.

Alongside this, there is likely to be greater cross-border and regional collaborations among European countries to lower the prices of new medicines during negotiations especially for cancer and orphan diseases (46, 111, 168–170, 207).

##### Valuing new cancer medicines

There have been initiatives by different stakeholder groups, including different cancer organizations, to try to quantify the level of benefit of new cancer medicines given ongoing funding concerns and little correlation to date between reimbursed prices and increased effectiveness (25, 208–212). However, there have been concerns with the use of surrogate markers such as response rates and disease free survival especially in patients with solid tumors (25, 39, 40, 179, 180). This has resulted in proposals to introduce more stringent assessment criteria such as minimal improvements in survival rates before granting premium prices for new cancer medicines (25, 34, 42, 213–216). Suggested minimal improvements include 3 to 6 months additional survival compared with current standards, although others have suggested less (215, 217). Alongside this, looking more critically at important factors for patients, and critically assessing the evidence alongside requested prices for decision making if only surrogate endpoint data is available (213, 216). Having said this, EUnetHTA believe surrogate endpoints in oncology are acceptable for accelerated or conditional marketing authorization approval; however, their utility for reimbursement and funding decisions will vary across countries (218).

There are also increased calls for moderation in the pricing of new cancer medicines as the continued increase in requested prices is becoming unsustainable (219–221). Such calls are enhanced by currently limited correlation between the degree of spend on cancer care and overall survival rates (222, 223). This is part of a general considerations on issues of fairer pricing with for instance the WHO recently launching the “Fair Price Forum” (158). There are also calls for proactive engagement by health authorities and governments in determining the potential initial price for a new cancer medicine based on the perceived health gain vs. current standards and improving negotiation skills during pricing discussions (111). Such discussions could also include whether the definition for orphan disease status needs refining into those medicines for genuine orphan diseases vs. companies using orphan disease status to increase requested prices for their new oncology medicines, which has resulted in very high prices especially for combinations (47).

Alongside this, there needs to be greater scrutiny regarding the existing spend on cancer medicines with greater understanding of opportunity costs across the different components of cancer care, as well as encouraging greater involvement of NGOs and others in helping to fund cancer medicines especially in LMICs, similar to the situation for treatments for HIV/ AIDS, cancer and NCDs (10, 105, 107, 128, 224, 225).

There also needs to be greater re-evaluation of reimbursed prices, including potential discounts, as more standard medicines in oncology become available as low cost generics or biosimilars. This is increasingly essential to fund increased volumes and new valued oncology medicines to improve care within finite resources.

#### Post Launch Activities

Post launch activities (Figure 1) include (i) monitoring the effectiveness and safety of new medicines in routine clinical practice using information in either patient registries or Electronic Health Records, (ii) evaluating ongoing MEAs and (iii) monitoring physicians prescribing against agreed guidance or quality indicators (3, 116, 141, 148).

Examples of studies monitoring the effectiveness and/ or safety of new medicines, as well as monitoring prescribing against agreed guidance, are contained in Table 2.

**Table 2:** Examples of post launch studies to improve future care provision.

**Country/Region and disease area**	**Summary of studies**
Belgium – patients with NVAF (226)	• The appropriateness of prescribing of either rivaroxaban or dabigatran in patients with non-valvular atrial fibrillation (NVAF) was evaluated, with the primary outcome measure being the prevalence of inappropriate prescribing • 69 patients were evaluated; 23% had one with an additional 26% more than one inappropriate criterion. The most frequent inappropriate criteria were inappropriate choices, wrong dose, and impractical mode of administration (26–28%) • The authors concluded that reinforcing education of health care professionals and patients is needed to improve future care
Catalonia (Spain) (227)—dabigatran in atrial fibrillation	• The study showed concerns with the appreciable number of patients over 80 years old receiving dabigatran for atrial fibrillation and not receiving the recommended dose • Renal function was also not being recorded in an appreciable number of patients (30%) and concerns that 17% of patients had previous ischemic heart disease which is a contraindication to dabigatran • Further educational initiatives are being planned to address this
Italy and Sweden—Dronedarone (228, 229)	• The authors evaluated how reimbursement of dronedarone impacted on the utilization of other antiarrhythmic drugs • In Sweden, the launch of dronedarone resulted in increased prescribing of antiarrhythmics without a variation in amiodarone use. It was different in Italy (Emilia Romagna Region), where the launch of dronedarone did not influence prescribing overall of antiarrhythmics or amiodarone • The authors believed limited impact on amiodarone prescribing was probably due to caution among physicians toward dronedarone in line with regulatory recommendations and safety warnings • These findings have been used to further develop a model in Sweden to evaluate the effectiveness, safety, and cost of new medicines in routine clinical care. This sequential model, using electronic health records and administrative health databases, can help to optimize the introduction of new medicines, providing direction to other regions and countries
Sweden ARTIS (Anti Rheumatic Therapies in Sweden) (230, 231)	• This comprehensive registry study has shown that (i) patients with rheumatoid arthritis treated with biological drugs are not at increased risk of invasive melanoma; (ii) patients with rheumatoid arthritis selected for TNF alpha inhibitors are not at increased overall risk for cancer but have an increased relative risk of invasive melanoma • The authors concluded that given the small increase in absolute risk, there is no shift in the overall risk-benefit balance of TNF alpha inhibitors in clinical practice; however care needs to be exercised in patients at high risk of melanoma
Italy—GISEA registry (232)	• Evaluation of 4-year retention rates of TNF alpha inhibitors among patients with long standing rheumatoid arthritis (RA) • The authors found that persistence was overall lower than 50%, with etanercept having the best retention rate • The principal positive predictor of patient adherence to TNF alpha inhibitors was the concomitant use of methotrexate
Brazil—Comparative effectiveness of adalimumab or etanercept for rheumatoid arthritis (233)	• This open prospective cohort study sought to evaluate the effectiveness and safety among patients with rheumatoid arthritis (RA) within the Brazilian Public Health System using a variety of different outcome measures • This study showed similar effectiveness between adalimumab and etanercept, with both treatments well-tolerated • Both treatments were more effective in RA patients who had better functionality at the start of treatment and had spent longer in education
Registry for patients with psoriasis including Psocare (Italy), Biobadaderm (Spain), and Clalit Health Services (Israel) (234)	• The objective of this large database study in Italy was to quantify the risk of infections including serious infections in patients prescribed TNF alpha inhibitors compared with non-biological therapies including methotrexate and cyclosporine • Overall 17,739 patients were included and 23,357.5 person-years of follow-up • The authors concluded that in current clinical practice, treatment with TNF alpha inhibitors was not associated with a higher risk of serious infections than treatment with non-biologic therapies such as methotrexate and cyclosporine
Brazil—Clinical effectiveness of different types of insulin in patients with Type 1 Diabetes (235)	• 580 patients were enrolled using data from the Brazilian National health Services database in Minas Gerais (Region in Brazil) • Overall there was no clinically significant difference in HbA1c levels between insulin glargine and NPH insulin • This was illustrated by the frequency of glycemic control being similar between the two groups and no statistically significant difference between controlled and still uncontrolled patient groups for all analyzed factors • The authors concluded there were limited clinical difference between the two insulins in routine clinical care, and this did not justify current appreciable price differences between the two insulins in Brazil
Sweden—Anti-Obesity medicines (236)	• Analysis of patient level data assessing the characteristics and utilization of patients prescribed various weight-loss drugs in Sweden showed there was limited persistence with weight loss treatments in routine clinical practice with over three quarters of patients continuing their treatment for <1 year • A high percentage of patients (28 to 32%) had a history of depression or antidepressant treatment which is a specific contraindication for rimonabant • Over 40% of patients on sibutramine had a history of hypertension and/or cardiovascular disease, which is a contraindication • Over a third of patients had no documented weight change after treatment
Brazil—Ten year follow up of kidney transplant patients receiving either cyclosporine or tacrolimus (237)	• This study involved 13,811 patients registered with the Brazilian National health Service database • Overall, a higher risk of graft loss was seen with tacrolimus vs. cyclosporine. Other factors increasing the risk of graft loss included kidney grafts coming from deceased donors and from more elderly patients, a median period of dialysis > 47 months before transplantation and a diagnosis of diabetes as the primary cause of chronic kidney disease • The authors concluded that tacrolimus-based regimens provided poorer outcomes against current beliefs and its use should be reviewed
Sweden—Effectiveness and adherence to second generation DAAs (238)	• This cross-sectional study involved national data from the Prescribed Drug Register and InfCare Hepatitis (quality register) • 3,447 patients in Sweden were initiated on second generation DAAs for Hepatitis C during 2014-2015 • The estimated overall cure rate was 96%, although there were some variations between genotypes
	• There was a high level of adherence to the introduction protocol (up to 94.2% for drug recommendations and 87% for treatment eligibility) • Overall, there was rapid uptake and equal distribution of DAAs in Sweden appreciably improving care for these patients
Adherence to national antibiotic guidelines in Namibia (239)	• The study found that the majority of prescriptions (over 60%) complied with national standard treatment guidelines (NSTGs) • However, compliance rates were lower than national targets (95%) which needs to be addressed. In addition, most prescriptions were empiric • There are ongoing moves to improve adherence to guidelines including developing quality indicators. Adherence to guidelines is also important in ambulatory care to improve future antibiotic use

*NB, DAA—Direct acting antivirals*.

It is likely that the use of real world data (RWD) will grow to support reimbursement and funding decisions with increasing use of electronic health records (183), enhanced in Europe through initiatives such as GetReal, which is a 3-year project involving the Innovative Medicines Initiative (IMI), pharmaceutical companies, academia, HTA agencies, payers, regulators, patient organizations as well as commercial organizations in generating patient level data (184). This is particularly important with medicines for orphan diseases where only limited data may be available at launch. However, ownership of RWD is a critical issue (108).

It is also likely that we will see greater historical data to help appraise the role and value of new medicines for orphan diseases with the full implementation of the European Reference Network (50, 52).

There are also ongoing activities particularly among LMICs to improve the activities and influence of drug and therapeutic committees (DTCs) to enhance the rational use of medicines especially in their hospitals as this can be variable (126, 240, 241). These activities can be enhanced by introducing legislation and other activities to increase the role of DTCs as currently seen in South Africa (240, 242). This builds on activities among Western countries to improve the rational use of medicines generally and post launch, including the interface (125). There is also ongoing research within Stockholm, Sweden, to document the impact of DTCs on influencing the use of new medicines given ongoing resource pressures. Overall Sweden, including Stockholm Country Council, has been one of the leading countries to co-ordinate such activities to improve the managed entry of new medicines, providing direction to other countries (136). This will continue.

Norway also provides an example of hospitals coming together for joint pricing negotiations for new and established medicines to improve affordability, for example low prices are now being paid for infliximab biosimilar and medicines for cancer (243). As mentioned, we are also seeing a growth in cross-border and regional collaborations to improve pricing negotiations for new medicines, which will continue (46, 168, 169, 207).

There is also ongoing research among European payers and their advisers to seek additional ways to enhance the rational use of medicines to help ensure available funds are prioritized for valued medicines. This together with the implications will be reported in future publications to further improve rational use.

Care is needed though when introducing new initiatives as expectations may not always be realized. This was seen in both Abu Dhabi and Korea when introducing policies to enhance competition and use of generics within their healthcare systems (244, 245).

### Multiple Criteria Decision Analysis (MCDA) Tools Especially for Patients With Cancer and Orphan Diseases

We are also seeing the growth in MCDA tools as a potential way to enhance structured, transparent and explicit approaches to reimbursement and funding decisions, resulting in guidance from different groups (33, 71, 246, 247). This also includes guidance to LMICs on potential ways to implement MCDAs to enhance transparency and standardization in funding decisions for new medicines (248). MCDA is an extension of decision theory that supports decision makers who have multiple (possibly conflicting) objectives by decomposing the decision objectives into key criteria (249). MCDA does not replace judgement, but rather identifies, collects and structures the information required by those making judgements to support the deliberative process.

Several public agencies and health insurers (such as G-BA in Germany, NICE in England and Wales and PBAC in Australia) are already using or proposing MCDA approaches in healthcare decision making, including prioritization of resources and activities (e.g., in Thailand), given concerns with uncertainty and rising prices, and this is likely to grow (247, 250–254). In most MCDAs, criteria such as disease severity, likely health outcomes including the safety profile, effectiveness, likely total costs/ budget impact, and activities surrounding the implementation of the new medicine, are mostly used to enhance decision making in addition to the quality of the evidence (33, 69, 255). Overall, it is increasingly believed MCDA can be used to support the HTA process; however, methodological challenges still remain (254, 256).

There are also ongoing initiatives, such as in the Netherlands and the UK, to use structured deliberative MCDA as opposed to the classic algorithmic MCDA in appraising new health technologies. In this approach, an in-depth consideration of a broad range of criteria takes place, including a critical assessment of available evidence. This requires relevant stakeholders to be explicit about their judgment regarding each criterion, and the impact on decisions. It is argued that structured deliberative MCDA improves the quality, consistency and transparency of decisions, and is likely to be applied more in the future. We will be monitoring these developments and their implications for reimbursement and funding decisions for new premium priced medicines.

It is perhaps not surprising that most MCDAs have been proposed for medicines for patients with cancer and orphan diseases given the level of uncertainty surrounding a number of these medicines, high requested prices, the heterogeneous nature of key stakeholders, and the emotive nature of the disease area (2, 67, 68, 72, 257). A number of the suggestions to improve reimbursement negotiations for new medicines for orphan diseases followed the controversy surrounding the reimbursement for enzyme replacement therapy for the symptomatic treatment of Fabry disease in the Netherlands at an incremental cost/ quality adjusted life-year of €3.3 million (37). A similar situation was seen with alglucosidase alfa to treat Pompe's disease. The estimated cost/ QALY was €0.3–0.9 million for the classic form of Pompe's disease up to €15 million/ QALY for the non-classic form (37). One of the principal MCDAs that evolved from this experience was the development of a Transparent Value Framework (TVF) for new medicines for orphan diseases (258–260) driven by MoCA (Mechanism for Co-ordinated Access to Orphan Medicinal Products). The TVF (Table 3) consists of four elements of value together with the extent to which each criterion is met, with the findings subsequently used as a possible basis for pricing negotiations (259, 260).

**Table 3:** The transparent value framework [adapted from (260)].

**Criterion**	**Low degree**	**Medium degree**	**High degree**
Available alternatives/Unmet Need	Alternatives are available and the new medicine does not address areas of unmet need	Alternatives are available but major unmet need still exist	There are currently no alternatives available and the ned medicine addresses major unmet need
(Relative) effectiveness—the degree of net health benefit relative to alternatives including no treatment. Net benefit includes the degree of health gain including improved Quality of Life (QoL) vs. potential side effects from the new medicine for orphan diseases	Incremental	Major	Curative
Response Rate – based on clinically relevant endpoints and time frames	<30%	30–60%	>60%
Degree of Certainty—based on available documentation (defined as the certainty of the claim made by the company)	Promising but not well-documented	Plausible	Unequivocal

It must be recognized though that the TVF is a non-prescriptive and non-binding value framework, which means that EU member states still have the responsibility for reimbursement decisions with regard to new medicines for orphan diseases in their country.

Having said this, the MoCA process is continuing with the TVF to provide a mechanism for joint discussions in this crucial area among all key stakeholder groups in Europe (259, 261) given current disparities in the availability of medicines for rare diseases across Europe (262). Such developments could also aid EURORDIS in its objective to have 3 to 5 times more therapies for rare diseases approved per year than currently by 2025 and three to five times cheaper to enhance access and affordability (50). This may be helped by improvements in genomics to aid diagnosis and discussions around fair pricing, potentially helped by joint purchasing and pricing negotiations, to re-balance current disparities in the availability of medicines for orphan diseases across countries (14, 47, 50, 169, 262). This can start with early dialogue between all key stakeholder groups as part of Horizon Scanning activities (section Pre-launch Activities), coupled with discussions on registries to improve information about orphan diseases and new medicines post launch. In addition, discussions about realistic pricing expectations among all key stakeholder groups starting pre-launch (49, 50, 263, 264).

### Possible Different Models for Financing New Medicines

New ways of addressing and financing new medicines are also being discussed given the extent of unmet need. A number of proposals have been suggested to finance R&D and new medicines given increasing concerns with issues of access and affordability (108). These include issues of acceptable value as well as de-linking the costs of R&D from a medicine's price, with basic research currently being predominantly undertaken in universities or funded by public sources (108, 265–268). De-linking models have been postulated for new cancer medicines in addition to new antimicrobials (267, 269). The situation with antibiotics is especially critical given the scarcity of new antibiotics in development to combat rising resistance rates with their appreciable impact on morbidity, mortality, and costs (270). The Drugs for Neglected Disease Partnership has been seen as a potential model for future de-linkage activities (108).

We are also seeing initiatives such as the Triple Helix model for innovation involving partnerships between universities, industry and governments (108, 271). An example of this is the development of new innovations in radiotherapy involving the Karolinska Institutet in Sweden together with other universities, private companies, and the government (108).

Potential new approaches also include a combination of push and pull incentives. Push mechanisms involve for instance direct investments in basic research and product development (272), which can include public sector research grants or research institutions (273). Pull mechanisms provide confidence in future sales and possible profitability through giving assurance on potential sales (272). Such approaches can stimulate research activities leading to the launch of new medicines in areas of need such as new medicines to treat rare orphan diseases or neglected diseases (272).

Health Impact Bonds (HIBs) have also been proposed, implemented for instance by a government determining suitable health programmes for finance and engaging intermediary stakeholders to help market potential investment opportunities (274). The programmes would be administered by healthcare providers, with external evaluators responsible for monitoring their progress (274). The first HIB was a program run in California for patients with asthma based on attaining over a 2-year period a 30% reduction in emergency room visits and a 50% reduction in asthma-related hospitalisations (274).

Additional possibilities include establishing a megafund as a single financial entity to invest in multiple biomedical projects (275). The intention is that by having one megafund, which entails a large portfolio of biomedical projects during different stages of drug development, the risk associated with attrition rates can be decreased to improve access and availability of new medicines. Such approaches have been proposed for new oncology medicines as well as medicines for rare diseases (275–277).

Lastly, annuity payments have been proposed for new gene therapies in view of their requested prices and limited uptake to date with such costs (47, 109, 278). We are already seeing companies offering installment payment plans to lung cancer patients to spread the costs (107).

There is ongoing research among payers and their advisers in Europe to discuss these and other potential approaches to improve financing and access to new valued medicines whilst maintaining sustainability of healthcare systems. This will be reported in the future.

We are aware that we have principally concentrated on the perspective of payers and their advisers. However, we believe this is appropriate especially among European and South American countries and Korea given their crucial role in the funding and utilization of new medicines.

## Conclusions

There is continued unmet need for new medicines especially for disease areas such as cancer, immunological diseases and orphan diseases. However, there are concerns with ever increasing prices along with funding increased medicine volumes with growing rates of patients with infectious diseases and NCDs worldwide. This requires co-ordinated activities by all key stakeholder groups to seek to fund new valued medicines whilst maintaining or attaining universal access to healthcare, with health considered a goal for all individuals. Co-ordinated activities among payers have been successful with limiting increases in expenditure in Europe (279); however, new approaches are now needed given increasing resource pressures alongside continual unmet need.

A number of approaches have been discussed to help fund new valued medicines overcoming current barriers. This includes developing new models to optimize the managed entry of new medicines starting up to 36 months before likely EMA approval through horizon scanning activities. In addition, more stringent assessment of the actual role and value of new medicines to ensure funding is directed to those new medicines that are the most valued in all or sub-populations, as well as greater involvement of patients in key areas. This will be closely monitored. As part of this, we are likely to see continued growth in MEAs to help with the financing of new medicines although there are concerns with these including continuing confidential discounts. This is likely to lead to increased discussions whether such discounts should continue within public healthcare systems. There are also likely to be clearer objectives around any proposed MEAs in the future as well as greater monitoring of their outcomes, including any administrative financial burden, to guide future schemes, and this will be followed up in future research projects.

We are also likely to see new initiatives especially in higher income countries around MCDAs for new medicines such as the TVF for orphan diseases and possibly new cancer medicines. Ongoing activities with the MoCA and EURODIS will also be closely monitored to see if this improves access to new medicines for orphan diseases in the future. Any MCDA for new cancer medicines are likely to include effectiveness/outcome criteria given current concerns with surrogate markers and limited additional overall survival for a number of new cancer medicines. Alongside this, there is likely to be increasing re-evaluation of prices and discounts for existing patented medicines, especially high priced medicines, once the comparators used in the evaluations become available as low cost generics or biosimilars. This will also be closely monitored given concerns with increasing expenditure on cancer medicines and issues of sustainability.

We are also likely to see continued growth in cross-border and regional collaborations to enhance pricing negotiations especially surrounding new medicines for orphan diseases (170). In addition, greater generation of real world evidence around new medicines given the continued growth in electronic healthcare systems to inform future treatment decisions. This will also be the subject of future research projects involving some of the co-authors. There will also continue to be ongoing research around different approaches to the financing of new medicines especially from the standpoint of payers and their advisers, and we will also be reporting on this in the near future.

The principal issue for a number of LMICs are concerns with the availability and access to essential medicines as defined by the WHO and others. This includes for instance ensuring increased access to appropriate medicines for patients with NCDs including cancer as well as enhancing their appropriate use, which involves enhancing adherence rates where these are a concern (10, 101, 225). Alongside this, accelerating moves toward universal health care. These are essential first steps before LMICs actively consider funding new treatments, unless these are funded by donors such as those for HIV and multidrug resistant TB, given the limited health gain with most new medicines. Alongside this, instigating improved systems management to reduce out-of-stock situations in public healthcare facilities, as well as greater accountability and transparency in decision making, building on current initiatives (81, 242). We will also be monitoring this in the future. We will also see the growth of HTAs among LMICs evaluating the cost-effectiveness and value for money of new interventions to improve evidence-based decision-making. This though will require legislation and policies as well as making suitable resources available (280).

We are already seeing DTC activities grow in LMICs to improve the quality and efficiency of prescribing, and this will continue alongside initiatives to improve physician education regarding the use medicines. As a result helping to reduce any inappropriate influence of pharmaceutical company activities (281). Alongside this, we are likely to see increased activities to monitor the appropriate use of medicines especially among LMICs given concerns with inappropriate prescribing leading to increased costs and for instance higher antimicrobial resistance rates (239, 240, 242). This is already happening for instance across Africa (282), and will increasingly include monitoring of prescribing against national treatment guidelines, and any other authoritative guidelines, building on current initiatives (239, 283).

There will also need to be greater scrutiny of the quality of medicines, especially generics, among a number of LMICs to improve their acceptance and use.

Moves to introduce fairer prices for medicines, including potential consortia, will also grow to improve access to key medicines as LMICs strive toward universal access, and this will also be the subject of future research projects.

## Author Contributions

BG, AB, PVB, WO, CR, AF, AHi, APM and SS contributed to the initial hypothesis for the paper. BG, AB, PVB, WO, CR, AF, SB, AHi, APM, SS, AK, MG, JG, AT, TB, AS, IH, RS, AM, AG, GP, ZMi, GA, OL, CS, GS, VK, JY, EM, RJ, MO, H-Y K, AJ, FK, JF, OM, MP, MW, VM-P, IM, DM, TN, JF, DT, CZ, ED, JM, RM, BW, ZMa, SC and AHa contributed to the final paper.

### Conflict of Interest Statement

The authors declare that the research was conducted in the absence of any commercial or financial relationships that could be construed as a potential conflict of interest.
